# Application of mNGS in the Etiological Diagnosis of Thoracic and Abdominal Infection in Patients With End-Stage Liver Disease

**DOI:** 10.3389/fcimb.2021.741220

**Published:** 2022-01-05

**Authors:** Hongmei Chen, Ye Zhang, Jie Zheng, Lei Shi, Yingli He, Yinghua Niu, Jine Lei, Yingren Zhao, Han Xia, Tianyan Chen

**Affiliations:** ^1^ Department of Infectious Diseases, The First Affiliated Hospital of Xi’an Jiaotong University, Xi’an, China; ^2^ Department of Scientific Affairs, Hugobiotech Co., Ltd., Beijing, China

**Keywords:** end-stage liver disease (ESLD), thoracic and abdominal infections, culture, metagenomic next-generation sequencing (mNGS), diagnosis criterion, neutrophil count

## Abstract

**Background:**

Despite the obvious advantages of metagenomic next-generation sequencing (mNGS) in etiological diagnosis of various infectious diseases, there are few reports on etiological diagnosis of suspected thoracic and abdominal infections in patients with end-stage liver disease (ESLD).

**Methods:**

Seventy-three ESLD patients were enrolled from January 2019 to May 2021 due to suspected complicated thoracic and abdominal infections with poor response to empirical anti-infective treatment. Pleural effusion and ascites samples of these patients were collected for mNGS detection and conventional pathogen culture. The application value of mNGS in etiological diagnosis of thoracic and abdominal infections in ESLD patients was finally evaluated.

**Results:**

A total of 96 pathogens were detected using mNGS method, including 47 bacteria, 32 viruses, 14 fungi, 2 *Mycobacterium tuberculosis*, and 1 parasite. The positive rate of mNGS reached 42.5%, which was significantly higher than that of conventional culture method (21.9%) (p = 0.008). Considering neutrophil counts, the overall positive rate of bacteria detection of both methods in Polymorphonuclear Neutrophils (PMN) ≥250/mm^3^ group was 64.3% and in PMN <250/mm^3^ group was 23.7%. Compared with the final clinical diagnosis, the agreement rate of mNGS in patients with positive bacteria detection and with suspected positive bacteria detection was 78.6% (11/14) and 44.4% (8/18), respectively. In addition, the agreement rate of mNGS was 66.7% (4/6, respectively) in patients with positive and suspected fungal detection. Interestingly, of the 11 patients with fungal detection, 5 had alcoholic liver disease, accounting for 45.5% of all patients with alcoholic liver disease. We also detected 32 strains of viruses using mNGS, mainly cytomegalovirus (62.5%).

**Conclusions:**

The mNGS method is a useful supplement to conventional culture methods, which performs a higher positive rate, higher sensitivity, and broader pathogen spectrum, especially for rare pathogens and those difficult to culture. For ESLD patients, mNGS has great prospects in early etiological diagnosis of thoracic and abdominal infections. In addition, the cutoff values for the diagnosis of bacterial infection (PMN ≥250/mm^3^) in the thoracic and abdominal cavities may need to be redefined.

## Introduction

End-stage liver disease (ESLD) refers to the late stage of liver disease caused by various liver injuries, mainly the decompensated stage of liver cirrhosis and liver failure due to various reasons (Kamath et al., 2007; Trebicka et al., 2021). The mortality of ESLD patients is extremely high, while concomitant infection is an important factor leading to an increased mortality risk (Asrani et al., 2019; Fiore et al., 2019; Verma et al., 2020; Falleti et al., 2021). Therefore, effective control of infection plays an important role in improving the success rate of treatment. Due to the impact of multiple factors such as portal hypertension, hypoproteinemia, and bacterial translocation, secondary thoracic and abdominal infection is the most common type of infection in ESLD patients, complicated with a large amount of pleural effusion and ascites (Gentilini et al., 2002; European Association for the Study of the L, 2010; Pericleous et al., 2016; Song, 2018). In most cases, the disease onset is insidious, lacking typical clinical symptoms such as fever, abdominal tenderness, or rebound pain (Dever and Sheikh, 2015). In addition, conventional bacterial and fungal culture methods have a very low positive rate, making early diagnosis, especially etiological diagnosis, extremely difficult (Hillebrand et al., 1996). Clinically, infection is often considered if the neutrophil counts in pleural effusion and ascites are ≥250 × 10^6^/L (Runyon and Hoefs, 1984). Even in the absence of a clearly identified pathogen, in most cases, empirical broad-spectrum antibiotic therapy would be given, leading to a higher possibility of developing drug-resistant bacteria and possible occurrence of secondary infections (Rimola et al., 2000; Bernal et al., 2010; Nanchal and Ahmad, 2016; European Association for the Study of the Liver, 2018). For patients with poor responses to empirical anti-infective treatment and still suspected of having thoracic and abdominal infections, the mortality risk will further increase in the absence of targeted treatment. Therefore, finding an efficient and specific method for early etiological diagnosis is the key to transform empirical treatment into targeted treatment in order to control the infections more effectively.

Currently, metagenomic next-generation sequencing (mNGS) is a rapidly developing technology for etiological diagnosis (Chiu and Miller, 2019). In infectious diseases such as central nervous system infection, blood infection, and lung infection, it plays an important role in etiological diagnosis, especially the identification of *Mycobacterium tuberculosis* and rare pathogens (Satta et al., 2018; Wilson et al., 2019; Crawford et al., 2020; Chen et al., 2021). However, there are few reports on the detection of pathogens responsible for thoracic and abdominal infections in ESLD patients. In this study, we retrospectively analyze 73 ESLD patients with suspected thoracic and abdominal infections, who were poorly responsive to empirical anti-infective treatment. In order to identify the pathogens, 73 pleural effusion and ascites samples of the patients were collected for both mNGS detection and conventional bacterial and fungal culture. Then, the results were comparatively studied to further evaluate the application value of mNGS for early etiological diagnosis of thoracic and abdominal infections in ESLD patients.

## Materials and Methods

### Patient Enrollment

We enrolled 84 inpatients with suspected complicated thoracic and abdominal infections who were admitted to the Department of Infectious Diseases, the First Affiliated Hospital of Xi’an Jiaotong University from January 2019 to May 2021. According to the diagnostic criteria of EASL Clinical Practice Guidelines for the Management of Patients With Decompensated Cirrhosis (2018 Edition) (European Association for the Study of the Liver, 2018), a total of 73 ESLD patients with suspected thoracic and abdominal infections who were poorly responsive to empirical anti-infective treatment were finally included in this paper. Pleural effusion and ascites samples (16 and 57, respectively) of these patients were collected for simultaneous mNGS detection and conventional bacterial and fungal culture.

**Figure 1:**
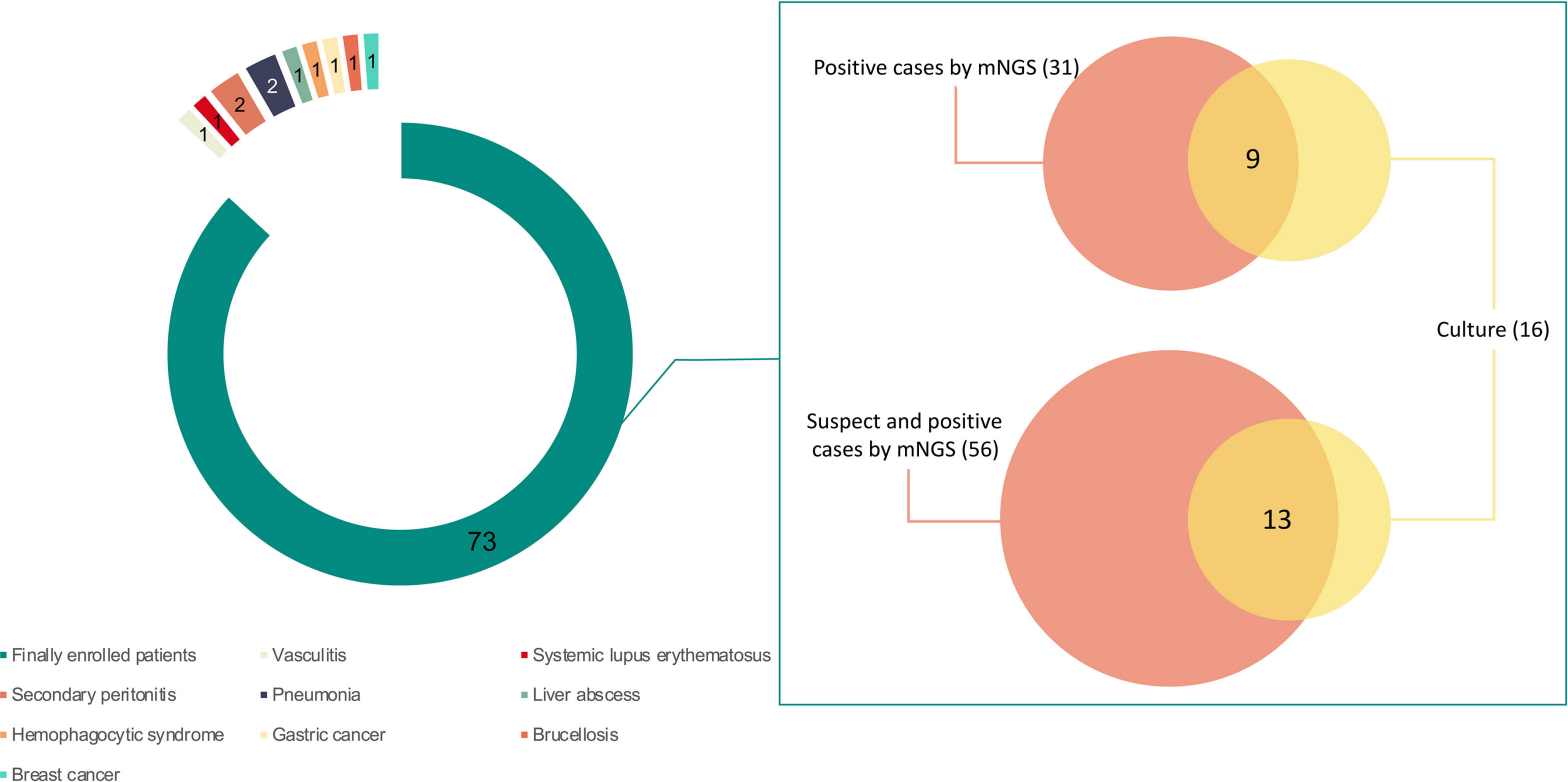
The enrolled patients and their detected results by metagenomic next-generation sequencing (mNGS) and culture. Seventy-three out of 84 patients were finally enrolled in this study. Sixteen patients were positive by culture. A total of 56 patients were detected with pathogens by mNGS, of whom 31 were considered as positive. Pathogens were found in 13 patients by both mNGS and culture, and 9 were considered as positive by both methods. Of the 9 patients with positive results, 7 were detected with bacteria consistent at the genus level and 5 were with bacteria consistent at the species level.

Eleven out of 84 patients were excluded, including two cases of peritonitis secondary to abdominal surgery, 1 case of gastric cancer, 1 case of breast cancer, 1 case of liver abscess, 2 cases of pneumonia, 1 case of brucellosis, 1 case of systemic lupus erythematosus, 1 case of vasculitis, and 1 case of hemophagocytic syndrome (**Figure 1**).

A poor response to empirical anti-infective treatment was considered when the patient had little improvement of clinical symptoms and had no significant decrease of pleural effusion/ascites or neutrophil counts after 3–5 days of antibiotic therapy.

### Isolate Identification by Culture

The clinical specimens were processed according to the recommended microbiological procedures. Species were identified through colony morphology, conventional biochemical reactions, and/or the use of an automated system (bioMerieux, Marcy l’Etoile).

### Metagenomic Next-Generation Sequencing Detection

After collection, the samples were transmitted on dry ice immediately to Hugobiotech Co., Ltd. (Beijing), for mNGS detection. DNA was extracted from all samples using QIAamp DNA Micro Kit (QIAGEN) according to the manual. QIAseq™ Ultralow Input Library Kit (Illumina) was then used to build the DNA libraries. To evaluate the quality of the libraries, Qubit (Thermo Fisher) and Agilent 2100 Bioanalyzer (Agilent Technologies) were used. Only the libraries with high quality were used and sequenced on Nextseq 550 platform (Illumina) using NextSeq 500/550 High Output Kit v2.5 (Illumina) at 75 cycles. A negative and positive control was set for each run of sequencing. The raw data were analyzed on PACEseq (Hugobiotech, Beijing). Short or low-quality reads were removed from the raw data. The human DNA was also filtered out after aligning to human reference database (hg38). The remaining clean reads were finally aligned to Microbial Genome Databases (ftp://ftp.ncbi.nlm.nih.gov/genomes/). For detected microbes, including bacteria (*Mycobacteria* excluded), fungi (*Cryptococcus* excluded), and parasites, a positive mNGS result was given when its coverage ranked top 10 of the same kind of microbes and absent in the negative control [“No template” control (NTC)] or when its ratio of Reads per million between sample and NTC (RPM_sample_/RPM_NTC_) > 10 if RPM_NTC_ ≠ 0. For viruses, *M. tuberculosis*, and *Cryptococcus*, a positive mNGS result was considered when at least 1 unique read was mapped to species level and absent in NTC or when RPM_NTC_ ≠ 0 and RPM_sample_/RPM_NTC_ > 5.

### Statistical Analysis

SPSS v25.0 was used to do chi-square test to analyze the difference of results between mNGS and culture. The detection of fungi in patients with alcoholic liver disease and other causes of ESLD was also calculated using chi-square test.

## Results

### Demographic and Clinical Characteristics

The 73 enrolled ESLD patients consisted of 49 males (67.1%) and 24 females (32.9%). Distribution of the causes of ESLD included 30 cases of hepatitis B virus (HBV) infection (41.1%), 6 cases of hepatitis C virus (HCV) infection (8.2%), 1 case of hepatitis E virus (HEV) infection (1.3%), 11 cases of alcoholic liver disease (15.1%), 4 cases of autoimmune liver disease (5.5%), and 21 cases of unknown causes (28.8%). A total of 57 ascites samples (78.1%) and 16 pleural effusion samples (21.9%) were collected. In pleural effusion and ascites examinations, neutrophil counts were ≥250/mm^3^ in 14 samples (19.2%) and <250/mm^3^ in 59 samples (80.8%) (**Table 1**).

**Table 1:** Demographic and clinical characteristics of the 73 patients.

Characteristic	Value
**Male sex-no. (%)**	49 (67.1%)
**Age**	
Age (Mean-years)	54.6
Distribution-no. (%)	
30–39 years	13 (17.8%)
40–49 years	11 (15.1%)
50–59 years	24 (32.9%)
60–69 years	15 (20.5%)
70–79 years	8 (11.0%)
80–89 years	2 (2.7%)
**Cause-no. (%)**	
HBV	30 (41.1%)
HCV	6 (8.2%)
HEV	1 (1.3%)
Alcoholic	11 (15.1%)
Autoimmune	4 (5.5%)
Unknown	21 (28.8%)
**Neutrophil count in pleural fluid and ascites no. (%)**	
≥250/mm^3^	14 (19.2%)
<250/mm^3^	59 (80.8%)
**Specimen type-no. (%)**	
Ascites	57 (78.1%)
Pleural fluid	16 (21.9%)

HBV, hepatitis B virus; HCV, hepatitis C virus; HEV, hepatitis E virus.

### Distribution of Pathogens Detected by Metagenomic Next-Generation Sequencing and Culture

**Figure 2:**
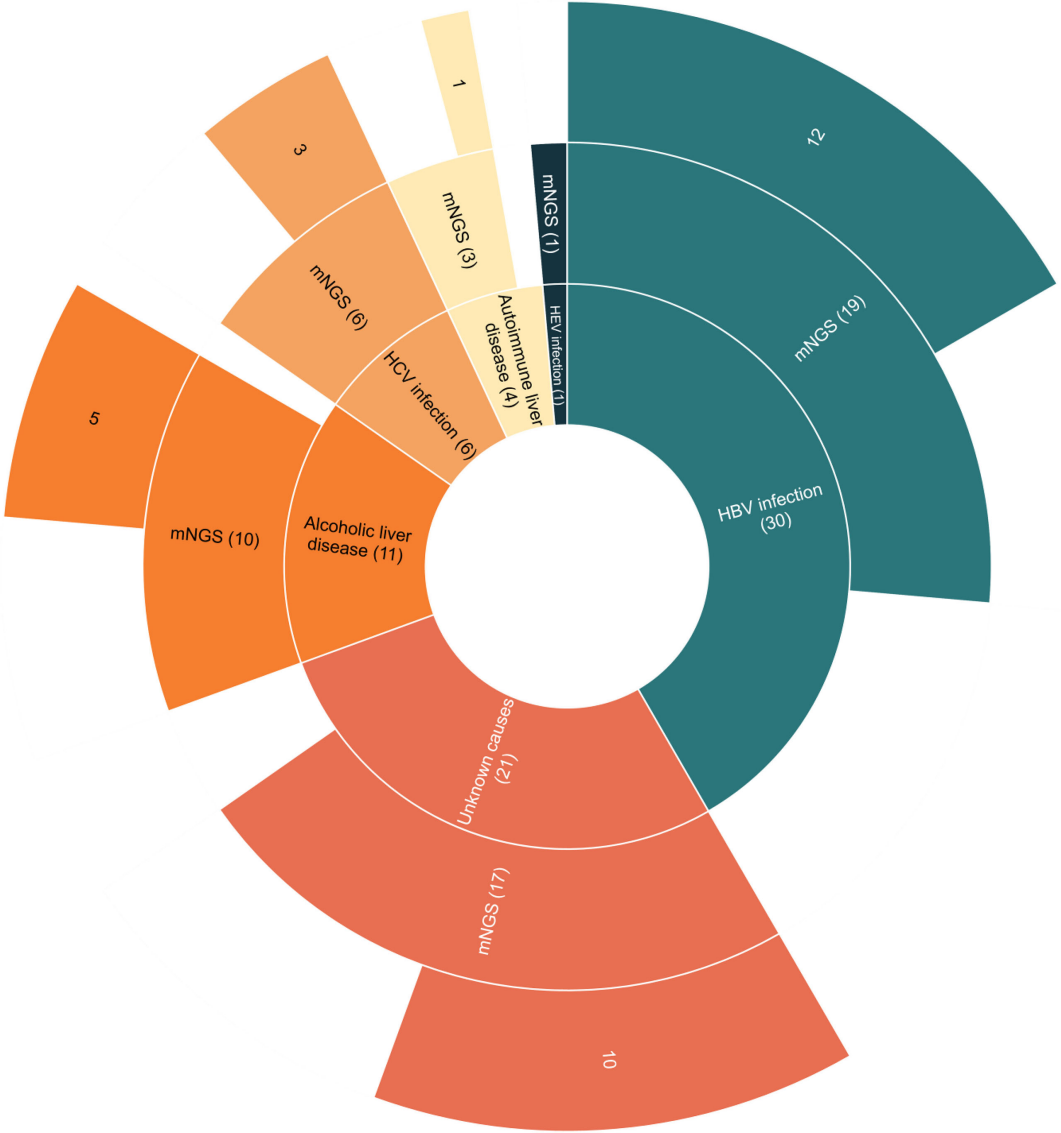
The metagenomic next-generation sequencing (mNGS) results of suspected thoracic and abdominal infection patients with different causes of end-stage liver disease (ESLD). Patients with ESLD of different causes were shown in the inner circle. Patients with detected pathogens by mNGS were shown in the middle circle. Patients with positive results by mNGS were shown in the outer circle.

**Figure 3:**
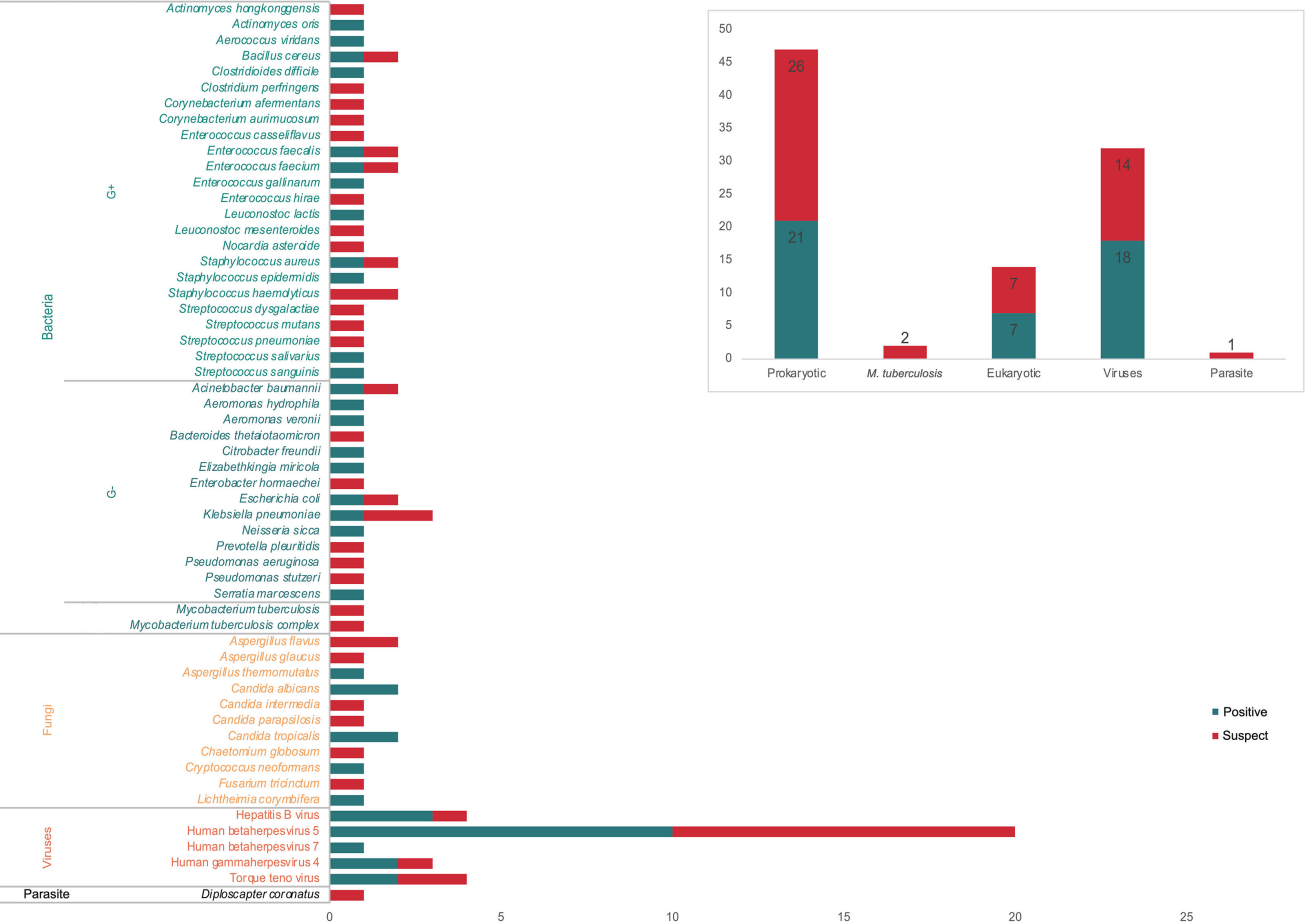
The positive and suspected pathogens detected by metagenomic next-generation sequencing (mNGS). A total of 96 pathogens were detected, including 47 bacteria, 32 viruses, 14 fungi, 2 *M. tuberculosis*, and 1 parasite, of which 46 were considered as positive.

**Figure 4:**
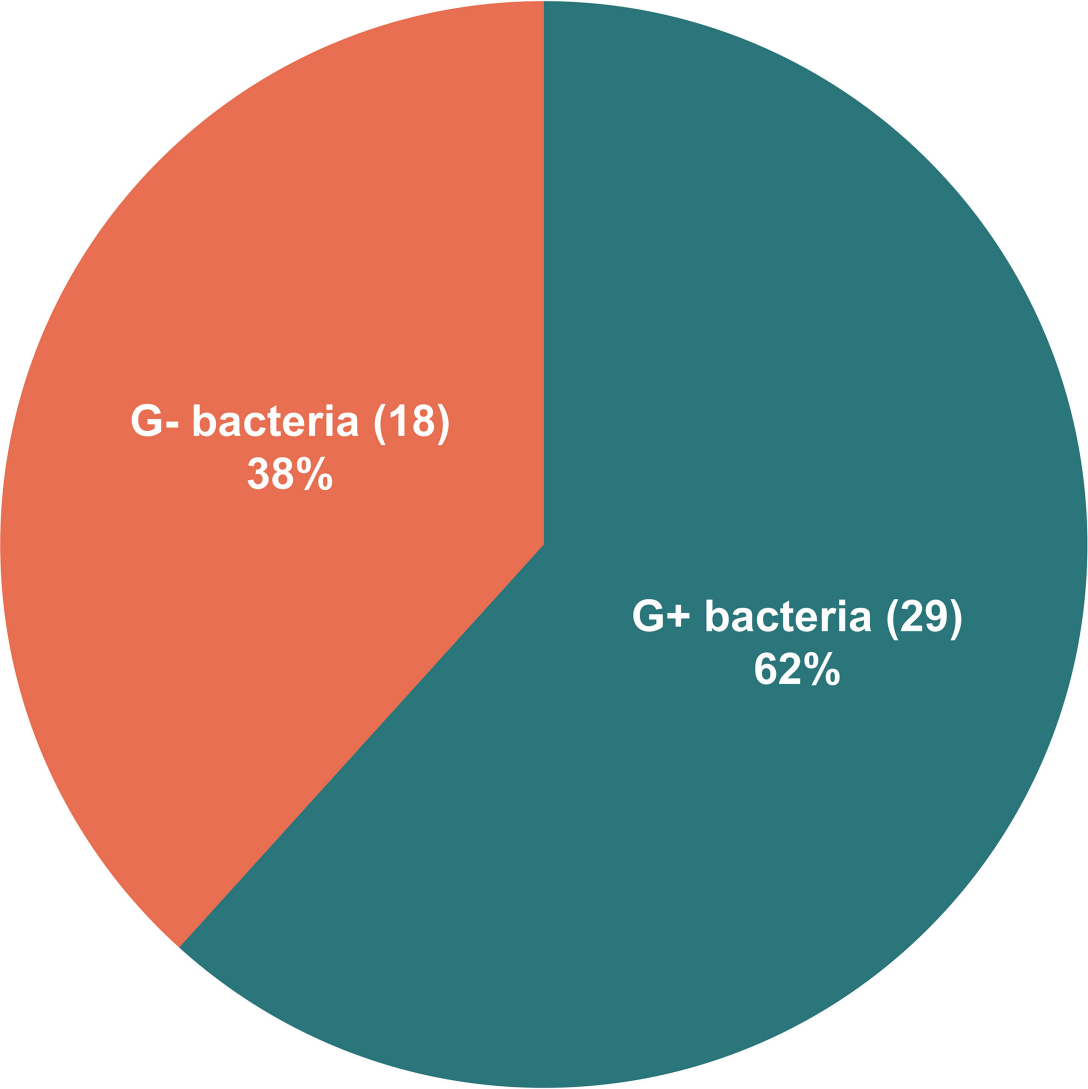
The distribution of Gram-positive bacteria and Gram-negative bacteria detected by metagenomic next-generation sequencing (mNGS). Of the 47 detected bacteria, 29 (62%) were Gram-positive bacteria and 18 (38%) were Gram-negative bacteria.

**Figure 5:**
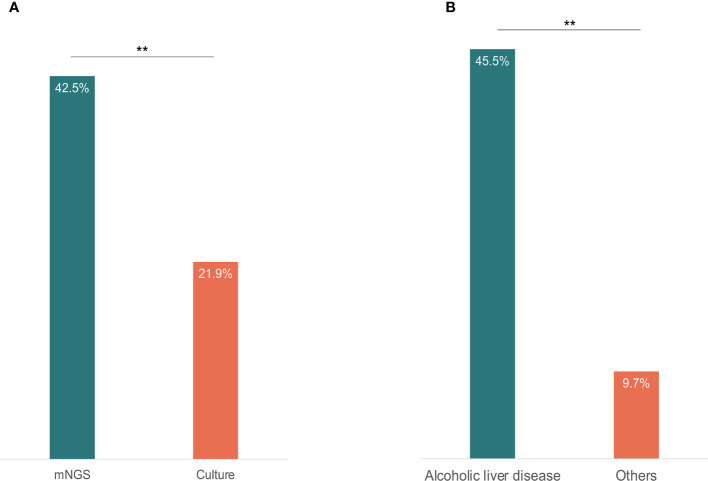
The content of positive results detected by mNGS and culture **(A)**, and the positive fungi results between alcoholic liver disease patients and other causes ESLD patients **(B)**. Chi-square test was used to analyze the difference of the comparisons. P< 0.01 was marked as **.

All the 73 samples were examined using mNGS method. The detailed information was shown in **Table S1**. A total of 31 samples (42.5%) were detected positive and 25 (34.2%) were suspected positive (**Figure 2**). A total of 96 pathogens were detected, including 46 positive pathogens and 50 suspected pathogens. There were 47 strains of bacteria, including 62% (29/47) Gram-positive bacteria and 38% (18/47) Gram-negative bacteria, 32 strains of viruses, 14 strains of fungi, 2 strains of *M. tuberculosis*, and 1 strain of parasite (**Figures 3**, **4**). Conventional culture method was also used in these samples, and 16 cases were positive (16/73, 21.9%). A total of 19 bacteria and 2 fungi were detected by culture. Chi-square test of paired samples between mNGS and culture was calculated; the positive rate of mNGS was significantly higher than that of culture (p = 0.008) (**Figure 5** and **Table S2**).

Nine patients were detected as positive by both mNGS and culture (**Figure 1**). The detected bacteria were consistent at the genus level in 7 patients and were consistent at the species level in 5 patients.

### Positive Etiological Diagnosis Rate of Samples in Different Neutrophil Count Groups

According to the Polymorphonuclear Neutrophils (PMN) ≥250/mm^3^ diagnostic criteria for clinical diagnosis of spontaneous bacterial peritonitis or pleurisy, the 73 ascites samples were divided into two groups. The overall positive rate of each group of bacterial detection by both methods was also calculated, showing 64.3% (9/14) in PMN ≥250/mm^3^ group and 23.7% (14/59) in PMN <250/mm^3^ group (**Table 2**).

**Table 2:** The positive results of mNGS and culture between patients with different PMN count levels.

	PMN ≥250/mm^3^	PMN ＜250/mm^3^
mNGS positive alone	3	5
Culture positive alone	3	6
Both positive	3	3
Overall positive rate	64.3% (9/14)	23.7% (14/59)

mNGS, metagenomic next-generation sequencing.

### Treatment Protocol Adjustment, Clinical Outcomes, and Agreement Rate in Patients With Positive Bacterial Detection Using Metagenomic Next-Generation Sequencing Method

A total of 21 strains of positive pathogens were detected by mNGS in 14 patients. Eight out of the 14 patients (57.1%) were finally confirmed with spontaneous bacterial peritonitis. The treatment protocol was adjusted in 12 patients (85.7%) based on mNGS results. No adjustment was made in the remaining 2 patients due to the coverage by the original antibiotic regimens. Except one deceased patient and 2 patients who were discharged due to critical illness/economics, the conditions of all remaining 11 patients improved (**Table S3**). The agreement rate between mNGS results and clinical findings reached 78.6% (11/14).

A total of 18 patients were detected with suspected bacterial infections by mNGS (**Table S3**). Thirteen of the patients (13/18, 72.2%) were finally confirmed as spontaneous bacterial peritonitis with clearly identified pathogens based on mNGS. Of these patients, 6 experienced improved conditions after adjusted therapeutic regimens. There were also 2 patients with improved conditions after continuing treatment without any adjustment due to coverage by the original antibiotic regimen. The agreement rate between mNGS suspected positive results and clinical findings was 44.4% (8/18).

### Distribution of Etiological Factors, Adjustment of Treatment Protocol, Clinical Outcomes, and Agreement Rate in Patients With Positive Fungal Detection Using Metagenomic Next-Generation Sequencing Method

A total of 14 fungal strains were detected in 11 patients, including 7 positive strains and 7 suspected strains. Of these patients, 5 had alcoholic liver disease, accounting for 45.5% (5/11) of all patients with alcoholic liver disease. The fungal detection rate in patients with alcoholic liver disease was significantly higher than that in patients of other causes (p = 0.009) (**Figure 5** and **Table S4**).

Six patients were detected with fungi infections by mNGS, of which 2 were consistent with fungal culture results. A total of seven fungal strains were identified. Four patients (4/6, 66.7%) were finally diagnosed with fungal peritonitis. After antifungal treatments, the conditions of 2 cases significantly improved without liver transplantation. Two cases were discharged without treatment due to critical illness/economics. The remaining 2 cases died (**Table S5**). The clinical agreement rate was 66.7% (4/6).

Six patients with seven suspected fungi were detected by mNGS, while conventional fungal culture results were all negative (**Table S5**). Five patients (5/6, 83.3%) were finally diagnosed with fungal peritonitis. After antifungal treatment, 4 cases significantly improved. One case was discharged without treatment due to critical illness/economics. The remaining one case improved without antifungal treatment, which was clinically inconsistent. The clinical agreement rate was 66.7% (4/6).

*M. tuberculosis* was detected in 2 patients, with only one specific sequence of each patient. One patient had ascites resulting from liver cirrhosis of unknown cause. After antituberculosis treatment for 1 month, the ascites did not subside significantly, and the drug was discontinued due to obvious liver injuries. The other patient had pleural effusion caused by liver cirrhosis due to HBV infection; this patient did not receive antituberculosis treatment because there was no other evidence of tuberculosis infection. After empirical anti-infective treatment, the pleural effusion gradually subsided. One strain of *Diploscapter coronata* was also detected in 1 patient with ascites resulting from liver cirrhosis of unknown cause, but the clinical relevance could not be determined because this critically ill patient gave up treatment and was discharged.

A total of 32 strains of viruses were detected, including 20 strains of cytomegalovirus that accounted for the majority, 4 strains of Torque teno virus, 4 strains of HBV, 3 strains of herpesvirus type 4, and 1 strain of herpesvirus type 7. Further research is needed to analyze the virus infection in ESLD patients.

## Discussion

In this study, we collected 73 pleural effusion and ascites samples from ESLD patients. The mNGS identified 31 positive cases (42.5%) and 25 suspected positive cases (34.2%). A total of 96 pathogens were detected, including 46 positive pathogens and 50 suspected pathogens. In comparison, conventional culture method identified 16 positive cases (21.9%), including 19 bacteria and 2 fungi. After chi-square test of paired samples, the positive rate of mNGS was significantly higher than that of culture (p = 0.008). As a highly sensitive detection method, mNGS has shown broad application prospects in pathogen diagnosis of infectious diseases (Chiu and Miller, 2019), as well as in this study, mNGS method demonstrated a higher positive rate, higher sensitivity, and broader pathogen spectrum in pathogen detection. In terms of etiological diagnosis of infectious diseases, mNGS is a useful supplement to the conventional culture method. In this study, the positive rate of culture was extremely low. One possible reason is that most ESLD cases of this study had a history of multiple antibody treatments before. This might greatly affect the detection rate of conventional culture method.

According to EASL Clinical Practice Guidelines for the Management of Patients With Decompensated Cirrhosis, the diagnosis of decompensated cirrhosis complicated with spontaneous bacterial peritonitis or pleurisy is mainly based on two objective criteria in clinical practice, including neutrophil counts in pleural effusion and ascites ≥250/mm^3^ and positive results of pathogen detection (European Association for the Study of the Liver, 2018). However, due to the low positive rate of conventional bacterial culture, the criterion of a clinical diagnosis of PMN ≥250/mm^3^ is often used in practice, followed by empirical antibiotic treatment (Hillebrand et al., 1996). Therefore, we also divided the 73 pleural effusion and ascites samples into 2 groups using PMN 250/mm^3^ as the cutoff value to calculate the positive rate of bacteria detection. The overall positive rate was 64.3% in PMN ≥250/mm^3^ group and 23.7% in PMN <250/mm^3^ group. Though the overall bacteria positive rate is much higher in PMN ≥250/mm^3^ group, the pathogens were still undetectable in 35.7% of the samples. In addition, bacteria were also detected in 23.7% of the PMN <250/mm^3^ group, who were considered without bacterial infections at first by the diagnosis criterion of PMN, causing difficulties in clinical decision-making and delayed timing of treatment. This indicated that the application of the state-of-the-art mNGS technology had significantly increased the positive detection rate of pathogens. On the other hand, PMN counts in pleural effusion and ascites were more affected, especially in ESLD patients who have received empiric antibiotic treatment. The results suggested that the diagnostic cutoff value of PMN ≥250/mm^3^ could not accurately determine the presence of spontaneous bacterial peritonitis or pleurisy. It is necessary to further expand the sample size to determine a more accurate clinical diagnostic cutoff value.

The mNGS results showed 47 bacteria from 31 patients, including 21 positive strains and 26 suspected strains. Gram-positive bacteria accounted for 62%, and Gram-negative bacteria accounted for 38%. This significantly differed from previous reports that thoracic and abdominal infections were dominated by Gram-negative bacteria (2016; Fiore et al., 2019). The possible reason is that these 73 pleural effusion and ascites samples were all obtained from ESLD patients, most of whom were in critical condition, repeatedly hospitalized, and frequently treated with broad-spectrum antibiotics. The empirical medication for thoracic and abdominal infections was mainly the third-generation cephalosporins and carbapenem antibiotics against Gram-negative bacteria (Fernandez et al., 2019). Considering the high cost of mNGS detection, the timing of our sample collection for mNGS detection was when the patients were unresponsive to empirical treatment in most cases. This might lead to an elevated detection rate of G+ bacteria after the selection by antibiotics against Gram-negative bacteria. It also suggested that when severe thoracic and abdominal infections were considered in critically ill ESLD patients, the timing of mNGS detection should be moved forward to reduce the impact of antibiotics in order to better support clinical decision-making.

Fungi are also important pathogens in ESLD patients complicated with thoracic and abdominal infections (Hu et al., 2019). Using the mNGS method, 14 fungi were detected in 11 patients with an overall positive rate of 15.1%. The positive rate of fungal culture was even lower. Only 2 cases (2.7%) were detected as positive with fungi infection among the 73 patients, both consistent with the mNGS results.

Compared with the final diagnostic results, the agreement rate of mNGS reached 78.6% and 44.4% in patients with positive bacteria and suspected bacteria results, respectively. The agreement rates of mNGS in patients with positive and suspected fungal detection were both 66.7%. It seemed that in ESLD patients who received empirical antibiotic treatment, the positive criteria of mNGS for bacterial and fungal detection were too strict, though more samples are needed for statistical analysis. Meanwhile, clinicians and laboratory specialists should work together to discuss more accurate interpretation rules.

Interestingly, of the 11 patients with positive fungal detection, 5 cases experienced alcoholic liver disease, accounting for 45.5% (5/11) of all patients with alcoholic liver disease. This indicated that ESLD patients with alcoholic liver disease experienced a significantly higher risk of fungal infection than those with other causes (p = 0.009). Some studies have shown that intestinal microecological imbalance is often accompanied by increased intestinal permeability in patients with alcoholic liver disease, whose composition of intestinal flora significantly differs from that of healthy people (Engen et al., 2015; Szabo, 2018). Yang et al. (2017) found that alcohol can relocate not only bacteria and their products but also fungi and their products in the intestines of mice. This may partially explain why fungal pathogens responsible for thoracic and abdominal infections are more common in patients with alcoholic liver disease.

In patients with ESLD, tuberculosis infection in the thoracic and abdominal cavity is not rare due to an impaired immune function (Sanai and Bzeizi, 2005). However, the limitation of culture conditions has made it more difficult to diagnose (Chow et al., 2002; Sanai and Bzeizi, 2005). For clinically highly suspected tuberculosis infections without a clear diagnostic basis, experimental antituberculosis treatment is often used for verification. However, ESLD patients usually have extremely poor liver reserves and are unable to withstand the side effects of antituberculosis drugs, causing noncompliance and treatment failure (Devarbhavi et al., 2013). The high sensitivity of mNGS and its independence on bacteria proliferation can greatly increase the possibility of clinical detection of certain rare pathogens and pathogens difficult to culture, such as *M. tuberculosis*.

Bacteria are the most common pathogens of thoracic and abdominal infections in ESLD patients. Conventional culture methods mainly detect bacteria, fungi, and *M. tuberculosis*, while viruses are rarely detected. Therefore, clinicians pay less attention to the presence of viral infections in thoracic and abdominal cavities. In this study, 32 strains of viruses were detected by mNGS, including 20 strains of cytomegalovirus, 4 strains of Torque teno virus, 4 strains of HBV, 3 strains of herpesvirus type 4, and 1 strain of herpesvirus type 7. It is well-known that cytomegalovirus and Epstein–Barr virus (EBV) infections are very common in transplant patients due to the use of immunosuppressants (Bunchorntavakul and Reddy, 2020). The ESLD patients with immune dysfunction may also be infected by these viruses as detected by mNGS. Further basic and clinical studies are needed to determine the presence of viral peritonitis and pleurisy in ESLD patients.

Certainly, as a brand-new technology, mNGS has contributed to milestone advancement in etiological diagnosis of infectious diseases, but this technology still has many aspects to be improved. Firstly, mNGS is highly sensitive, its detected pathogen spectrum is significantly broader than that of conventional culture methods, but the clinical interpretation still lacks a unified standard. Laboratory specialists and clinicians need to work together to determine whether the detected microorganisms have clinical importance. So, more samples are also needed for data analysis and formulation of the interpretation rules. Secondly, mNGS uses gene sequencing method for pathogen detection; it can neither determine the biological activity of pathogens nor perform drug sensitivity tests like conventional culture methods. In addition, this study also has some shortcomings. Because of the cost of mNGS, most of the examined patients were critically ill and had the economic burden. The timing of mNGS detection was mostly when the patients were unresponsive to the empirical treatment rather than before the treatment. Previous treatment regimens may have a certain impact on both mNGS and conventional culture. The number of cases in this study was relatively small. More samples are needed to further determine the value of mNGS in the etiological diagnosis of thoracic and abdominal infections.

In summary, mNGS method produced a higher positive rate and sensitivity of pathogen detection and a broader pathogen spectrum in ESLD patients with thoracic and abdominal infections. The mNGS method is a useful supplement to conventional methods and has great application prospects in early etiological diagnosis of thoracic and abdominal infections. In addition, the cutoff values of PMN ≥250/mm^3^ for the diagnosis of bacterial infection in the thoracic and abdominal cavities need to be redefined. Compared with the final diagnosis, both positive and suspected bacterial and fungal pathogens detected by mNGS have a high clinical agreement rate. This suggests that in ESLD patients receiving empirical antibiotic treatment, the positive criteria for defining bacteria and fungi in pleural effusion and ascites samples using mNGS method might be extended.

## Data Availability Statement

The data presented in the study are deposited in the National Genomics Data Center repository (https://www.cncb.ac.cn/), accession number PRJCA006182.

## Ethics Statement

The studies involving human participants were reviewed and approved by the ethical review committee of the First Affiliated Hospital of Xi’an Jiaotong University. The patients/participants provided their written informed consent to participate in this study. Written informed consent was obtained from the individual(s) for the publication of any potentially identifiable images or data included in this article.

## Author Contributions

HC and TC designed and drafted the paper. HC collaborated in the collection of medical records. HC, LS, YH, YN, and JL were involved in the clinical care and management of the patients. JZ and YRZ did the statistical analysis. HX analyzed the mNGS data. HC and YZ revised the article. All authors read and approved the final article. All authors contributed to the article and approved the submitted version.

## Funding

This research was supported by the National Natural Science Foundation of China (Grant 82070641), the Clinical Research Award of the First Affiliated Hospital of Xi’an Jiaotong University, China (No. XJTU1AF-CRF-2018-002), the National Natural Science Foundation of Shaanxi Province (2018JM7146), and the Science and Technology Project of Xi’an (21RGSF0013). The funding sources had no involvement in the study design, writing the article, or decision to submit for publication.

## Conflict of Interest

YZ and HX are employed by Hugobiotech Co., Ltd.

The remaining authors declare that the research was conducted in the absence of any commercial relations or financial relationships of interest that might be a conflict of interest.

## Publisher’s Note

All claims expressed in this article are solely those of the authors and do not necessarily represent those of their affiliated organizations, or those of the publisher, the editors and the reviewers. Any product that may be evaluated in this article, or claim that may be made by its manufacturer, is not guaranteed or endorsed by the publisher.
